# Methods for collecting and rearing three sympatric biocontrol agents of *Adelges tsugae* (Hemiptera: Adelgidae) in eastern North America

**DOI:** 10.1093/jisesa/ieaf107

**Published:** 2025-12-19

**Authors:** Elizabeth M D’Auria, Nicholas J Dietschler, Tonya D Bittner, Isis A L Caetano, Mark C Whitmore

**Affiliations:** Department of Natural Resources and the Environment, Cornell University, Ithaca, NY, USA; Department of Natural Resources and the Environment, Cornell University, Ithaca, NY, USA; Department of Ecology and Evolutionary Biology, Cornell University, Ithaca, NY, USA; Department of Natural Resources and the Environment, Cornell University, Ithaca, NY, USA; Department of Natural Resources and the Environment, Cornell University, Ithaca, NY, USA; Department of Natural Resources and the Environment, Cornell University, Ithaca, NY, USA

**Keywords:** invasive species, classical biological control, insect rearing, pest management

## Abstract

A decline in global forest health, due in part to the spread of insect pests, has prompted research into effective, sustainable, and economically feasible solutions to manage forest pests, including the use of classical biological control. Among the top 5 imperiled tree species in North America, eastern hemlock (*Tsuga canadensis* (L.) Carriere) is impacted by the invasive hemlock woolly adelgid, *Adelges tsugae* Annand, accidentally introduced from southern Japan. Three specialist natural enemies of *A. tsugae* found on western hemlock woolly adelgid in western North America have been deemed promising biological control agents and approved for release—2 species of silver fly, *Leucotaraxis argenticollis* (Zetterstedt) and *Leucotaraxis piniperda* (Malloch), and a beetle, *Laricobius nigrinus* Fender. Supplying quantities of these predators needed for research and release has special challenges given their dietary and habitat needs. We have developed novel collection and rearing methods for producing these 3 predators simultaneously in large numbers as part of a rearing and release strategy in eastern North America.

## Introduction

The Intergovernmental Science-Policy Platform on Biodiversity and Ecosystem Services (IPBES) calculated the cost of invasive species to the global economy to be $423 billion annually (Roy et al., 2024). Insects are perhaps the most pervasive invaders, leading to declines in forest health globally (Venette and Hutchison 2021), requiring research into effective, sustainable, and comprehensive management solutions. One powerful long-term solution is classical biological control (CBC), which involves the intentional introduction of natural enemies to suppress invasive pest populations (Wright 2014, reviewed in Hajek and Eilenberg 2018). CBC strategies have been used successfully since the introduction of the vedalia beetle *Novius* (=*Rodolia) cardinalis* (Mulsant) (Coleoptera: Coccinellidae) and the parasitic fly *Chryptochaetum iceryae* Williston (Diptera: Cryptochetidae) in 1888 to manage cottony cushion scale, *Icerya purchasi* Maskell (Hemiptera: Monophlebidae) in California citrus (Caltagirone and Doutt 1989). Since then, there have been many examples of success in combating invasive insect pests with mass rearing and release of natural enemies. For example, a decline in winter moth (*Operophtera brumata* (L.) [Lepidoptera: Geometridae]) density has been attributed to the successful establishment of the tachinid parasitoid *Cyzenis albicans* Fallén (Diptera: Tachinidae) at 41 of the 44 release sites in the Northeastern United States, causing up to 40% parasitism (Elkinton et al. 2021). However, a challenge of CBC is sourcing large quantities of biological control agents needed to study their life history and deploy for operational field releases.

Wild collection and laboratory mass rearing are the primary ways of supplying CBC agents, and each method has a unique set of challenges. Both strategies require dedicated facilities, specialized methods, and sourcing of the agents. Laboratory rearing requires continuous access to food, including prey for predators or an appropriate artificial diet. Additionally, the closed population can become inbred and cause a reduction in genetic variation and fitness or performance (Sørensen et al. 2012). Sourcing agents from the wild can reduce costs and preserve genetic variation but requires locating ephemeral communities of prey and their predators. The latter strategy is being tested for the hemlock woolly adelgid (HWA), *Adelges tsugae* Annand (Hemiptera: Adelgidae), a serious pest of eastern hemlock (*Tsuga canadensis* (L.) Carriere [Pinales: Pinaceae]) and Carolina hemlock (*T. caroliniana* Engelmann), across eastern North America (reviewed in Limbu et al. 2018).

First observed infesting trees in 1951 in Richmond, Virginia (Stoetzel et al. 2002), *A. tsugae* has caused widespread decline and mortality of hemlocks in eastern North America. Hemlocks are a foundation species whose disappearance can disrupt and alter the surrounding ecosystem by changing soil properties and species composition (Jenkins et al. 1999, Ellison et al. 2018). In the introduced eastern North American range, *A. tsugae* undergoes a complex polymorphic life cycle consisting of 2 parthenogenic generations per year (McClure 1989, Havill and Foottit 2007). The overwintering generation (sistens) includes an obligatory summer aestivation before development begins in fall, with adults maturing to lay eggs in late-winter or early-spring. The subsequent generation (progrediens) develops rapidly in spring or early summer, with their offspring restarting the cycle. Only the first nymphal instar (crawler) of each generation are mobile and can be spread over long distances by wind, animals, or humans (Limbu et al. 2018). Because of its parthenogenic reproduction, high fecundity, and passive dispersal, *A. tsugae* remains a challenge to manage (Limbu et al. 2018).

There have been efforts to manage *A. tsugae* through a diverse set of tactics such as chemical, silvicultural, and biological control (Mayfield 2020, 2023a). While effective, chemical treatments are costly and require repeated applications that are challenging at the landscape scale. Similarly, silvicultural practices such as creating canopy gaps to increase light levels improve the health of infested hemlock trees (Mayfield et al. 2023a); however, research on landscape-scale implementation and efficacy is lacking. Thus, implementing a biological control program offers a sustainable long-term solution for reaching remote locations through population growth and dispersal.

Worldwide, there are 11 species of hemlock (*Tsuga* spp.). Nine have a native coevolved association with *A. tsugae*, with only eastern and Carolina hemlocks lacking native adelgids (Havill et al. 2016). In western North America, there is a native *A. tsugae* lineage that feeds on western and mountain hemlock (*Tsuga heterophylla* and *Tsuga mertensiana*, Havill et al. 2016). Predator exclusion studies suggest *A. tsugae* populations in the native western range are reduced to low density by predators (Crandall et al. 2022), including 2 species of silver fly, *Leucotaraxis argenticollis* (Zetterstedt) (Diptera: Chamaemyiidae) and *Leucotaraxis piniperda* (Malloch), and a beetle, *Laricobius nigrinus* Fender (Coleoptera: Derodontidae), that specialize on *A. tsugae* (Kohler et al. 2008a, Grubin et al. 2011). These 3 sympatric natural enemies of *A. tsugae* have been deemed promising biological control agents and approved for release in the eastern United States (reviewed in Mayfield et al. 2023b). Despite the widespread establishment of *La. nigrinus* (Mausel et al. 2010, Crandall et al. 2023) and significant predation of the overwintering generation in the introduced range (Jubb et al. 2020), populations of *A. tsugae* can quickly rebound due to their density-dependent parthenogenic life history (Crandall et al. 2020). The occurrence of *Leucotaraxis* spp. larval feeding during both *A. tsugae* generations suggests they provide complementary predation, potentially increasing biological control efficacy (Kohler et al. 2016, Rose et al. 2020). Therefore, *Le. argenticollis* and *Le. piniperda* are good candidates for applying continuous predation pressure covering all *A. tsugae* developmental stages (Mayfield et al. 2023b).

The main challenges of rearing *A. tsugae* biological control agents are sourcing and maintaining and housing the insects for use in research and release. Rearing a single generation of *Leucotaraxis* spp. can be done (Dietschler et al. 2023), but maintaining a colony is currently infeasible due to challenges of sustaining prey resources and phenological synchrony in the lab. Maintenance of a *La. nigrinus* colony has proven more successful but can also encounter issues with prey sourcing, predator survival, and synchrony (Foley et al. 2021). We have developed a novel composite rearing method consisting of annual wild collection and lab rearing of all 3 sympatric predators. In this paper, we (1) describe the sourcing of wild-collected predators, (2) assess collection timing that maximizes all 3 predator species, and (3) evaluate a novel *La. nigrinus* rearing method.

## Materials and Methods

### Sourcing Predators

Predator collections occurred annually in the native HWA western North American range from 2018 through 2025. Branches of *A. tsugae*-infested western hemlock were pruned and shipped to the insect biocontainment facility at Cornell University using USDA APHIS PPQ-approved methods (permit #P526P-21-00588 and P526P-21-00589) as described in Dietschler et al. (2021). For each collection, infested boughs (∼0.5 m length) were placed into 42-gal (163 l), 3 mil thick, black plastic trash bags, double bagged, and sealed into a double-walled cardboard shipping box and shipped via overnight delivery. Collections were made between the end of January and July, with variation in timing and duration between years (Table 1). Because heavy infestations containing abundant predators are ephemeral from year to year, collection sites are found by surveying annually. Early-season collections were generally smaller and were observed for predator load to determine which collection sites to revisit later in the season for optimal predator production.

**Table 1.** Total predators collected from western North American collections from 2018 to 2025 by year and species

Year	Collection date range	*Leucotaraxis* spp.	*Le. argenticollis* ^a^	*Le. piniperda* ^a^	*La. nigrinus* prepupae	*La. nigrinus* adults^b^	*La. nigrinus % eclosion*
**2018**	24 Mar to 29 May	2,280	–	–	1,338	82	6%
**2019**	19 Feb to 31 Jul	10,316	–	–	4,818	1,179	24%
**2020**	13 Feb to 27 May	10,095	–	–	9,677	3,899	40%
**2021**	30 Jan to 19 May	13,103	–	–	25,731	10,696	42%
**2022**	24 Jan to 24 May	12,382	2,805	9,538	25,227	12,135	48%
**2023**	30 Jan to 11 Jul	20,893	3,129	17,564	17,726	11,735	66%
**2024**	24 Feb to 13 May	48,661	3,743	44,639	15,558	10,507	67%
**2025**	19 Feb to 19 May	24,145	12,855	11,290	8,805	NA^c^	NA
**Total**		141,875	22,543	83,054	108,880	50,233	

a Species-specific totals do not equal combined *Leucotaraxis* totals, due to a portion of a collection not being identified.

b Adult *Laricobius nigrinus* represent wild-collected prepupae that completed development and emerge as adults in the lab.

c 2025 fall emergence of adult *La. nigrinus* had not occurred at the time of publication.

Initially, the target species were the 2 *Leucotaraxis* spp., because *La. nigrinus* was being lab reared from a colony using established methods (Foley et al. 2021). While piloting *La. nigrinus* rearing methods using wild beetle larvae present on collected western foliage, we observed large numbers of *La. nigrinus* prepupae dropping from foliage and began developing lab rearing methods in 2018 and 2019 to utilize this resource. This influenced a transition from colony-based rearing to developing hybrid methods consisting of wild collection and conventional lab rearing to utilize incidentally collected *La. nigrinus* larvae from western foliage. We will refer to this strategy as “composite rearing.” We continued to fine-tune methods each year to increase survival, and those methods are reflected here.

Sourcing predators for biological control from the same continent makes the logistics of collecting relatively easier than sourcing overseas. Nevertheless, extensive biocontainment precautions are used in working with western foliage in eastern North America to eliminate the introduction of non-target organisms. Western *A. tsugae* eggs and crawlers are present in the cages during collection of *La. nigrinus* fourth larval instar (prepupae), so several precautions were taken, discussed below, to exclude crawlers from those daily collections. Parasitoids of *Leucotaraxis* species in the genera *Pachyneuron* (Walker) (Hymenoptera: Pteromalidae) and *Melanips* (Walker) (Hymenoptera: Figitidae) have been identified from western collections (Kohler et al. 2008b, Celis et al. 2022). Only adult *Leucotaraxis* are permitted to leave biocontainment since this life stage harbors no parasitoids. Unlike *Leucotaraxis*, *La*. *nigrinus* has no known parasitoids, but we release them as adults to synchronize properly with HWA development.

### Collection Maintenance and Predator Emergence

The Sarkaria Arthropod Research Laboratory in Ithaca, NY is a USDA-certified insect biocontainment facility. The handling of non-native insects and the sanitation of equipment are guided by standard operating procedures of the biocontainment facility. The facility houses a greenhouse where we store wild-collected western hemlock foliage. Adult *Leucotaraxis* spp. emergence has been linked to heating degree day accumulation (Dietschler et al. 2021), so greenhouse temperatures are kept at 15.5 °C before being raised to 20 °C in mid-March to increase *Leucotaraxis* spp. emergence rates to maximize release numbers during the optimal (adult) life stages of HWA.

The biocontainment greenhouse was equipped with 90 custom acrylic cages (Leigh-Dale Specialties, Syracuse, NY), including 30 small cages (45.72 × 45.72 × 50.8 cm) and 60 large cages (69.85 × 50.8 × 45.72 cm). All cages were fitted with 120-µm nylon mesh windows (Component Supply Co., Sparta, TN) for ventilation while preventing HWA crawlers from escaping. Each large cage was fitted with 2 shoe boxes measuring 11.43 × 33.66 × 18.42 cm, and each small cage was fitted with 1 shoe box filled with saturated floral foam. In 2020, 1-in cork feet were added to each corner of the shoe boxes in large cages, enabling better access for *La. nigrinus* larval collection.

**Fig. 1.**
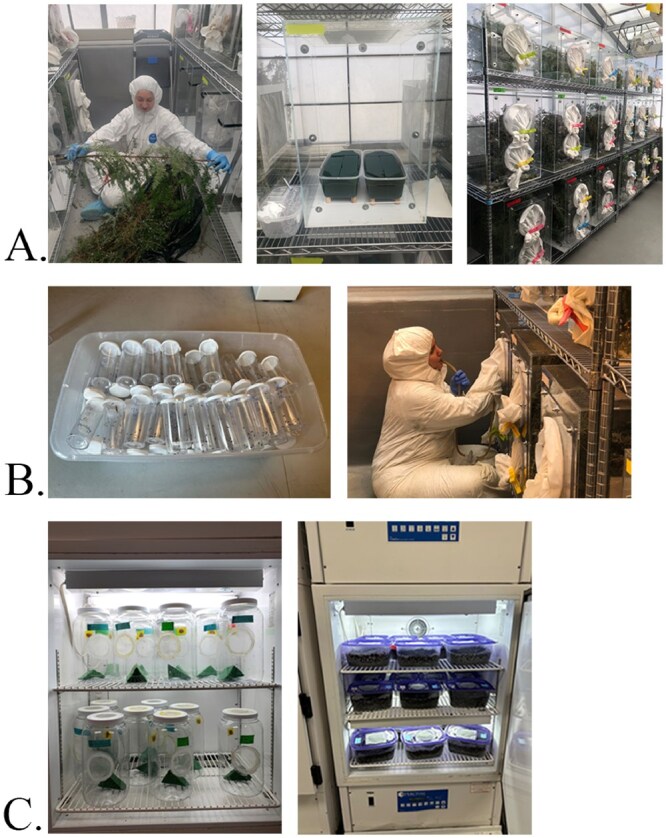
A) Unpacking western collected *Adelges tsugae*-infested foliage into cages prepared with cork-bolstered shoeboxes fitted with saturated foam brick. B) Predators are collected daily with an aspirator and transferred into vials for counting and processing. C) *Leucotaraxis* are temporarily held in jars before release and *Laricobius* prepupae are kept to aestivate before emerging as adults in the fall.

When a shipment was received, hemlock stems were removed from the bags and trimmed with hand pruners at a 45-degree angle 1 to 2 in. from the end of the stem (Fig. 1A). The stems were pushed into the hydrated floral foam bricks. Each shoe box fits approximately 15 stems of varying sizes. Floral foam was watered ad libitum. Once insect emergence had finished, the foliage was autoclaved and discarded.

Cages were checked daily, exploiting each predator’s natural behavior, for the presence of positively phototaxic adult *Leucotaraxis* at the top of cages and fourth instar *La. nigrinus* larvae (=prepupae) that dropped (“larval drop”) to cage floors searching for subterranean pupation sites. *Laricobius nigrinus* prepupae were collected in the morning and *Leucotaraxis* spp. in the afternoon when they were more actively emerging, using greenhouse lights to help aggregate flies at the top of cages. All taxa were grouped by cage number (which links to collection site) and date. All non-target generalist predators (eg spiders, lacewings) were collected daily and freeze-killed to protect our target species. All organisms were collected using an aspirator with a 9-dram plastic vial attached (Fig. 1B).

### Predator Processing and Rearing

The general operational pattern differs between *Leucotaraxis* and *Laricobius*. Adult flies are used for field release or research immediately in late winter through spring, generally being used within 2 weeks of emergence for best survival. Adult flies are released during the adult egg-laying stages of both *A. tsugae* generations. *Laricobius* beetles collected as prepupae in the spring must undergo a mandatory summer aestivation, synchronized with HWA, and emerge in the fall before being released as adults on the developing *A. tsugae* overwintering sistens generation. A small number of adult *La. nigrinus* are present in the western foliage when it is collected in spring. They are assumed to have expended most of their eggs, but we release them locally.

In the lab, *La. nigrinus* pupation containers were prepared following established protocols (Salom et al. 2012, Foley et al. 2021). Briefly, storage containers (10.16 × 20.64 × 15.24 cm) with ventilated mesh lids were prepared with a depth of approximately 7.5 cm soil media consisting of 4:1 peat:sand and 30% to 35% moisture (reverse-osmosis purified water) as measured by a HydroSense II soil moisture reader (Campbell Scientific, Logan, UT). Prepupae were sorted using a fine-tip paintbrush, and healthy prepupae were transferred to the pupation container. We limited each container to approximately 150 prepupae. Live prepupae were recorded by cage and date and tracked at collection-site level. The moisture level of the containers was maintained by weight throughout aestivation. In August, beetles are less susceptible to desiccation, so we let the moisture levels drop to 90% of the initial weight by gradual evaporation. Beetles were held in growth chambers (Percival Scientific Inc., Perry, IA, United States), and we followed Foley et al. (2021) guidelines for adjusting the temperatures seasonally (Fig. 1C). Initially set at 13 °C day temperature, 10 °C night temperature and a 12L:12D light schedule throughout the late spring, we increased the temperature to 19 °C day and night and 16L:8D light schedule in the summer.

Flies were counted and identified to species using male genitalia or pronotal setae (Dietschler et al. 2021, Gaimari and Havill 2021). These *Leucotaraxis* species emerge in temporally distinct species clusters in even sex ratios, making identification of males an effective way to quickly separate species (Dietschler et al. 2021, 2023); however, females can be identified with pronotal setae if males are not available. Flies were stored before use at 15 °C during the daylight period (6 AM to 8 PM) and 10 °C at night in plastic one-gal (3.78 l) jars supplied with a thumbprint-sized drop of 1:1 ratio honey:wheast on yellow label tape (Lacewing and Ladybug Food (Wheast, Planet Naturals, Bozeman, MT)) and a small saturated floral foam brick for hydration (Fig. 1C).

### *Laricobius nigrinus* Adult Emergence

In mid-August, adult *La. nigrinus* began to emerge from the soil and were collected daily through early December, when emergence ended. Field releases began in late September or early October after *A. tsugae* breaks aestivation (Dietschler et al. 2024). Additionally, at this time, we systematically lowered temperatures in the growth chambers to mimic fall temperatures (13 °C day, 10 °C night, and 12L:12D). Emerging adults are less sensitive to desiccation; therefore, water was only added when the soil surface was visibly dry. Collected adults were held temporarily in one-gal (3.78 l) plastic jars filled with locally sourced infested eastern hemlock branchlets set in moistened floral foam bricks wrapped in parafilm. They were held at 4 to 5 °C for up to 2 weeks until released or used in experiments.

### Data Analysis

We assessed annual predator collection trends, correlations, and *La. nigrinus* survival to adult. Predator emergence was binned by the month that foliage was collected from western North America, and predator abundance was expressed as per cage at the site level for each collection. Collected foliage was randomly assigned to cages by site, so cage size and position in the greenhouse were not taken into consideration. Adult *Leucotaraxis* spp. emergence was evaluated from 2018 to 2025 to determine patterns of emergence throughout the collection season. Emergence of *Le. argenticollis* and *Le. piniperda* was evaluated separately from 2022 to 2025, since methods to reliably identify and separate species at the time of emergence were developed in 2021 (Dietschler et al. 2021). *Laricobius nigrinus* collections were assessed for only the 2020 to 2025 seasons, after lab collection and rearing methods were fully developed. To assess *La. nigrinus* survival by collection month, we only included pupation containers where all the prepupae came from foliage collected in the same month; all containers with mixed collections were omitted from the analysis.

Predator abundance by sites and years was assessed for 2022 to 2025 with Spearman’s ranked correlation, comparing adult *Le. argenticollis* to *Le. piniperda* and *La. nigrinus* prepupae to both adult *Leucotaraxis* spp. *Leucotaraxis* spp. emergence data were log-transformed to reduce skewness and improve homogeneity of variance for *Leucotaraxis* spp. (ln +1) and both *Le. argenticollis* and *Le. piniperda* (ln) for ANOVA, and post-hoc Tukey HSD was used to compare emergence among collection months. Log transformation did not resolve distribution and variance issues for *La. nigrinus* prepupal collection data, so a non-parametric Kruskal–Wallis test and post-hoc pairwise Dunn test were used. We report corrected (Holm-Bonferroni) and uncorrected post-hoc analysis results, since *P*-value corrections are overly conservative. Cumulative annual survival of *La. nigrinus* larvae to adult and survival by collection month were assessed by binomial logistic regression and back-transformed to survival probability. Statistical analyses were performed in R, version 4.3.1 (R Core Team 2023). The *rstatix* package was used to run Kruskal–Wallace and Dunn tests (Kassambara 2023), *lme4* and *emmeans* package for binomial logistic regression (Bates et al. 2015, Lenth 2023), and *ggplot2* was used to create plots (Wickham 2016).

## Results

From 2018 through 2024, there was a general increase in collection numbers for *Leucotaraxis* spp., and after 2020, a stable output of adult *Laricobius* as rearing capacity (cage and shelf space) was increased and maximized in 2020 (Table 1). Collection timing varied by year, with all collections occurring between 24 January and 31 July (Supplementary Table S1). There was a significant positive correlation between the 2 *Leucotaraxis* spp. adults (rho = 0.324, S = 19,783, *P *= 0.015) and between *La. nigrinus* prepupae and *Le. argenticollis* (rho = 0.479, S = 15,238, *P* ≤ 0.001) and *Le. piniperda* (rho = 0.394, S = 17,737, *P *= 0.003), supporting that all 3 species can be efficiently targeted in collections (Supplementary Fig. S1).

### *Leucotaraxis* Spp. Collections

**Fig. 2.**
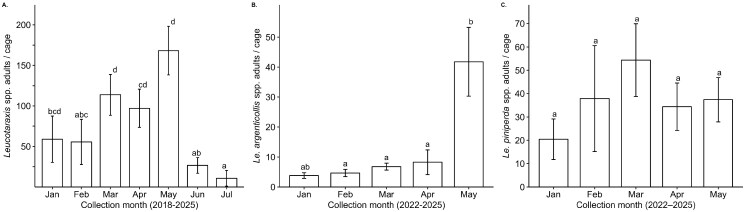
Pairwise comparison of mean adult *Leucotaraxis* spp. emergence by foliage collection month from 2018 to 2025 (A); and mean adult *Le. argenticollis* (B) and *Le. piniperda* (C) emergence from 2022 to 2025. Plots present raw data means and standard errors, and letters represent Tukey-HSD pairwise comparisons of log-transformed data for both *Leucotaraxis* species separately (ln(x)) and combined *Leucotaraxis* spp. (ln[x + 1]).

Adult emergence of *Leucotaraxis* spp. differed throughout the season over all years by collection month (*F*_6,292_ = 2.485, *P *= 0.02, Fig. 2A, Supplementary Table S2). Early-season collections in February showed lower adult emergence compared to those in March and May. Peak collection season is March to May, with adult fly collection numbers dropping in June and July, representing the end of collection season. January shows little difference compared to other collection months, but this collection period is only represented in a few years (2021 to 2023) from a small number of sites (*n* = 9), representing lower collection effort when compared to other months (Fig. 2A and Supplementary Table S1). Adult emergence of *Le. argenticollis* differed based on field collection month (*F*_4,101_ = 8.29, *P* ≤ 0.001, Fig. 2B, Supplementary Table S3), while *Le. piniperda* did not (*F*_4,99_ = 0.49, *P *= 0.75, Fig. 2C, Supplementary Table S4).

### *Laricobius nigrinus* Collections and Rearing Survival

**Fig. 3.**
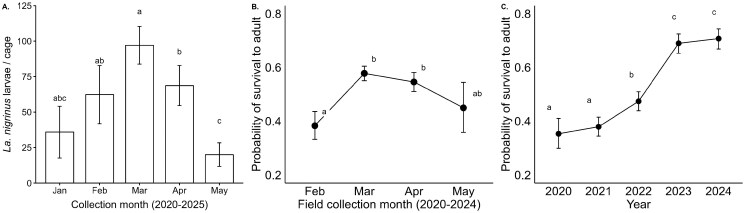
*Laricobius nigrinus* prepupal drop collection abundance from 2020 to 2025 (A). The probability that a collected *La. nigrinus* prepupae will survive to adult based on the month it was collected from 2020 to 2024 (B). The probability of *La. nigrinus* prepupal survival to adult by year (C). Each vertical bar represents site level means and standard error, letters show non-corrected pairwise comparison (α = 0.05) (A). See Supplementary Table S5 for Holm–Bonferroni correction. Points indicate estimated marginal means from back-transformed binomial logistic regressions and 95% confidence intervals (B and C).

*Laricobius nigrinus* prepupae abundance differed by field collection month from 2020 to 2025 (*H*_4,221_ = 23.795, *P* ≤ 0.001), as expected based on *La. nigrinus* synchrony with *A. tsugae* (Zilahi-Balogh et al. 2003). Prepupae were present in collections from January through May, with abundance building in January and February, peaking in March, decreasing in April, and finishing in May (Fig. 3A and Supplementary Table S5). Beetle prepupae are present at low numbers in June, but late-season collections were mostly discontinued by 2020. The largest differences in abundance were observed between pre/peak season (Feb to Apr) and late season (May) collections, with a marginal difference between pre-season survey collections in February and peak-season in March (Supplementary Table S5). Those collected in February have the lowest rates of survival to adult, with the highest survival being from March and April collections. Survival from May collections shows downward trends, with March survival being marginally higher than May (*P *= 0.053), but low abundance of May-collected prepupae reduced the number of pupation containers in the analysis (Fig. 3B and Supplementary Tables S5 and S6). Over years, cumulative survival of *La. nigrinus* to adult has significantly increased, with survival <38% in 2020 and 2021, increasing to 45% in 2022, and stabilizing just below 70% in 2023 and 2024 (Fig. 3C and Supplementary Table S7).

## Discussion

Beginning in 2018, we developed and refined methods for collecting and rearing 3 biological control agents for the management of *A. tsugae*, mitigating some challenges of maintaining year-round laboratory colonies. We collect heavily infested foliage annually that has been shown to contain the highest predator densities (AU2 in review), and positive correlations support that all 3 predators can be effectively targeted without tradeoffs in abundance among predator species. The increased fly abundance we observed later in the season (Mar to May) was influenced by eliminating unproductive collection sites earlier in the season (Jan to Feb, Fig. 2A), leading to March through May producing the greatest abundance of all 3 predators (Fig. 2A). Aside from requiring specialized facilities, the main challenge of this approach is that populations of *A. tsugae* in the native western range fluctuate annually at each site, requiring yearly surveys to locate collection sites (Mayfield et al. 2023b).

Employing what is known about the ecology of silver flies increases the effectiveness of using this wild collection strategy. For example, *Le. argenticollis* was the least abundant predator in collections (2022 to 2024, Table 1), and targeting sites with this species is a priority. Since *Le. argenticollis* overwinters as puparia and emerge in 2 distinct peaks (Dietschler et al. 2021, 2023), we can return to sites where this species was abundant during early collections, increasing the number of *Le. argenticollis* obtained during the second annual peak, occurring in May collections (Fig. 2B). In contrast, *Le. piniperda* showed no difference in emergence throughout the season (Fig. 2C). Evidence indicates *Le. piniperda* overwinter as larvae (Dietschler et al. 2023, NJD in review), and their single annual emergence could be more influenced by temperature-dependent development (Dietschler et al. 2021). Therefore, if *Le. piniperda* larvae are present on foliage, they are likely to complete development in the cages no matter when the collection occurred. Microclimate variation among collection sites adds variation in development and flattens the distribution of lab emergence.

The predictable temporal stratification of specialist predator emergence facilitates the efficient separation of *Leucotaraxis* flies by species (Dietschler et al. 2021). Identifying the *Leucotaraxis* spp. as they emerge from the cages and grouping them separately by species streamlines research planning and field release. It also allows for more single-species releases of known quantities, which aids in elucidating factors for successful population establishment.

For many years, *La. nigrinus* was a focus of the federal biological control program for *A. tsugae* (Mayfield et al. 2023b). Through 2019, we maintained a laboratory colony of *La. nigrinus* on infested eastern hemlock, which resulted in low adult survival, whereas wild-collected *La. nigrinus* prepupae were consistently present on collected western foliage and survived to adulthood at greater rates (IALC, unpublished data). At the same time, supplying the colony with adequate prey on eastern hemlock was becoming more difficult due to factors like HWA overwintering mortality (McAvoy et al., 2017) and declining tree health. The composite rearing program was fully implemented by 2020.

The life cycle of *La. nigrinus* is well-documented, and collection trends reported here are consistent with larval beetle feeding synchronized with *A. tsugae* oviposition and beetle prepupae dropping in March and April (Fig. 2A) (Zilahi-Balogh et al. 2003, Mausel et al. 2008). Notably, prepupae from foliage collected in March and April were more likely to survive to adult (Fig. 3B). This pattern of survival is best explained through collection timing synchronization with *La. nigrinus* natural life history, with larval development in these groups being more advanced at the time of collection and most feeding having already occurred in their natural environment. Younger larvae may be more susceptible to stress from collection, transport, and/or greenhouse conditions, whereas larvae collected late in the season may represent individuals that had less food available as HWA egg-laying decreases. Our composite rearing methods improved over time; colony-based rearing survival reported from other labs of 40% (Foley et al. 2021) was attained in the first 2 years, increasing to a current survival of approximately 70% (Fig. 3C). Our *La. nigrinus* rearing methods have been consistent since 2020, although 3 improvements have been made, including (1) being more selective to include only the healthiest larvae, (2) improved colony hygiene, and (3) improved soil moisture management. *La. nigrinus* pupation containers are maintained at seasonally specified temperature, daylength, and moisture level.

Repeated wild predator collections over the season, which are housed in the controlled warmer biocontainment greenhouse, increase insect development and emergence and enable better synchronization with local prey phenology throughout HWA’s eastern introduced range. The relatively cool temperatures over the winter–spring western collection season mean each batch of predators remains at earlier developmental stages than those previously collected and reared in warmer lab temperatures (Dietschler et al. 2021). This extends the overall fly collection season to allow for synchronization of adult fly releases with prey eggs across the invaded eastern range. Geographic and interannual variation in eastern *A. tsugae* oviposition timing is well documented, with onset ranging from early February to late April (McClure 1987, Gray and Salom 1996, Joseph et al. 2011, Dietschler et al. 2024), with improvements in phenological modeling offering better forecasting of important developmental stages (Crimmins et al. 2020).

There are few, if any, examples of biocontrol insect predator programs where the agents are repeatedly wild-collected rather than reared in colonies. Notably, the cottony cushion scale predator, *Novius* (=*Rodolia*) *cardinalis*, was successfully dispersed from a field insectary by transporting branches to new citrus groves (Caltagirone and Doutt 1989); and most recently, the parasitic fly, *C. albicans*, that has successfully controlled winter moth, *O. brumata*, in Massachusetts, was repeatedly sourced from populations of parasitized late-instar moth larvae on Vancouver Island, British Columbia, until establishment occurred locally. The methods we developed for wild collection were driven by serious challenges for rearing *Leucotaraxis* spp., long-lived insects that live on prey in trees, in a laboratory. Artificial diets have been attempted for other HWA predators, but these are minimally effective for short-term use (Cohen and Cheah 2015). While annual wild collecting is time- and resource-intensive for a few months, in this case, it offers efficiency through collecting 3 predators in tandem while preserving genetic diversity, avoiding potential fitness or performance issues (Sørensen et al. 2012). The addition of composite rearing of *La. nigrinus* eliminates the need to collect local-infested host tree material, which has become increasingly difficult as hemlock trees either get chemically treated or succumb to pest pressure. Moreover, composite rearing also substantially decreased the amount of time needed for maintenance of the beetles. A similar approach to collecting biocontrol agents could work in other systems where significant challenges for colony maintenance exist.

Future research on *Laricobius* may aim to uncover the mechanisms that influence the survival of western-collected *Laricobius* spp in the lab in order to further improve quantities. However, beetles are already establishing and dispersing naturally in the United States, with some high-abundance sites allowing for fall collection and distribution to new sites (Crandall et al. 2023, Mayfield et al. 2023b). The need for composite rearing programs is currently driven mainly by demand for *Leucotaraxis spp.* In the near term, research to better understand community dynamics in western populations may help target *Leucotaraxis* spp. more effectively.

By making collections throughout the season, all species are collected in abundance for research and release. Releasing these biological control predators could facilitate predator–prey equilibrium, managing *A. tsugae* populations and preventing tree mortality. Development of collection and rearing techniques that yield the entire suite of specialist predators has led to efficient mass production facilitating both laboratory and field research, with the goal of establishing effective and self-sustaining top-down control of HWA to reduce hemlock mortality in eastern North America.

## Supplementary Material

ieaf107_Supplementary_Data
