# Supplementary material for: Methods for collecting and rearing three sympatric biocontrol agents of Adelges tsugae (Hemiptera: Adelgidae) in eastern North America
Source: J Insect Sci. 2025 Dec 19;25(6):ieaf107. doi: 10.1093/jisesa/ieaf107 (PMC12715311; doi:10.1093/jisesa/ieaf107)
Supplement: ieaf107_Supplementary_Data [file ieaf107_supplementary_data.docx]

Supplemental Materials

| Year | Foliage Collection date ranges | *La. nigrinus* larval collection ranges | *Leucotaraxis spp.* collection ranges | SPID |
| --- | --- | --- | --- | --- |
| 2018 | 24-Mar to 29-May | 13-Apr to 4-May | 3-Apr to 25-Jun | no |
| 2019 | 19-Feb to 31-Jul | 14-Mar to 23-May | 25-Feb to 15-Aug | no |
| 2020 | 13-Feb to 27-May | 4-Mar to 19-May | 24-Feb to 23-Jul | no |
| 2021 | 30-Jan to 19-May | 17-Feb to 1-Jun | 10-Feb to 28-Jun | no |
| 2022 | 24-Jan to 24-May | 7-Feb to 8-May | 28-Jan to 22-Jun | yes |
| 2023 | 30-Jan to 11-Jul | 17-Feb to 30-May | 9-Feb to 30-Jun | yes |
| 2024 | 24-Feb to 13-May | 29-Feb to 26-May | 29 Feb to 24-Jun | yes |
| 2025 | 19-Feb to 19-May | 3-Mar to 26-May | 28 Feb to 23 Jun | yes |

Table S1. Date ranges for foliage and insect collections by year. “SPID” refers to whether the *Leucotaraxis* collections were identified to species throughout the season.


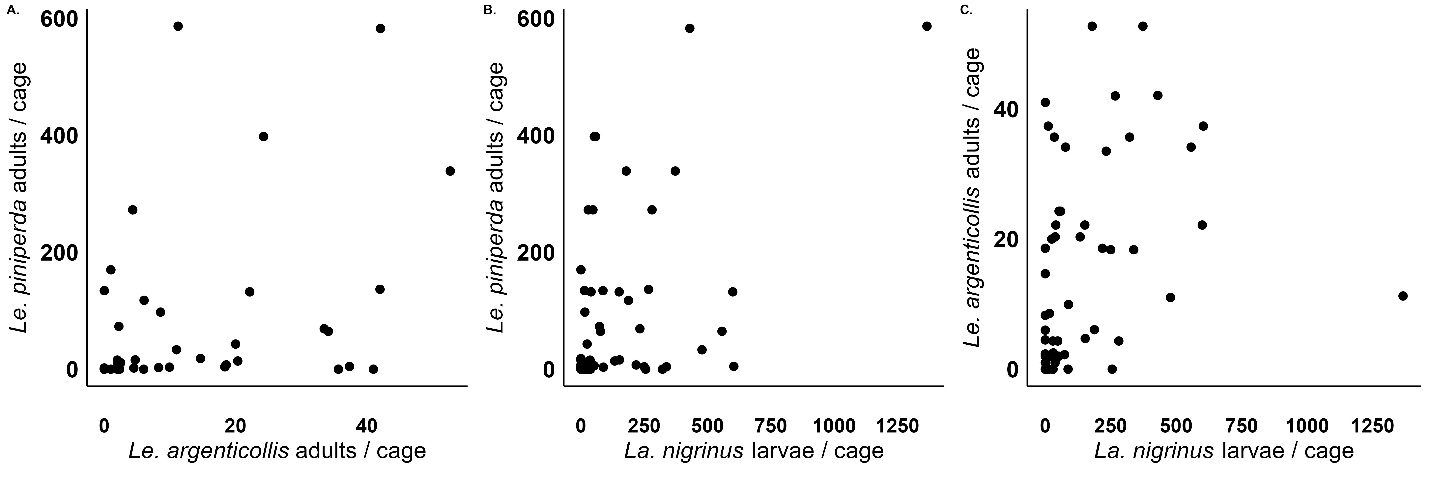


Figure S1: Spearman correlation comparison (2022-2025) of *Le. piniperda* and *Le. argenticollis* adults (A), *Le. piniperda* adults and *La. nigrinus* prepupae (B), and *Le. argenticollis* and *La. nigrinus* prepupae (C).

| Pairwise comparison | | Estimate | p value |
| --- | --- | --- | --- |
| Month 1 | Month 2 |  |  |
| *Leucotaraxis* spp. (2018-2025) | | | |
| January | February | -0.7326 | 0.896 |
| January | March | 0.2838 | 0.999 |
| January | April | -0.1769 | 1.0 |
| January | May | 0.7463 | 0.888 |
| January | June | -1.389 | 0.367 |
| January | July | -2.4461 | **0.041** |
| February | March | 1.0165 | **0.012** |
| February | April | 0.5557 | 0.266 |
| February | May | 1.479 | **0.004** |
| February | June | -0.6563 | 0.698 |
| February | July | -1.7135 | 0.08 |
| March | April | -0.4607 | 0.666 |
| March | May | 0.4625 | 0.717 |
| March | June | -1.6728 | **<0.001** |
| March | July | -2.73 | **<0.001** |
| April | May | 0.9232 | 0.548 |
| April | June | -1.2121 | **0.049** |
| April | July | -2.2693 | **0.004** |
| May | June | -2.1353 | **<0.001** |
| May | July | -3.1925 | **<0.001** |
| June | July | -1.0572 | 0.693 |

**Table S2:** Post-hoc Tukey’s HSD pairwise comparison of *Leucotaraxis* spp. collection month, from 2018-2025.

| Pairwise comparison | | Estimate | p value |
| --- | --- | --- | --- |
| Month 1 | Month 2 |  |  |
| *Leucotaraxis* *argenticollis* (2022-2025) | | | |
| January | February | 0.8397 | 1.0 |
| January | March | 2.9567 | 1.0 |
| January | April | 4.4311 | 0.999 |
| January | May | 37.9483 | 0.124 |
| February | March | 2.1169 | 0.999 |
| February | April | 3.5913 | 0.995 |
| February | May | 37.1085 | **0.001** |
| March | April | 1.4744 | 1.0 |
| March | May | 34.9916 | <0.001 |
| April | May | 33.5172 | **<0.001** |

**Table S3:** Post-hoc Tukey’s HSD pairwise comparison of *Leucotaraxis argenticollis*. collection month, from 2022-2025.

| Pairwise comparison | | Estimate | p value |
| --- | --- | --- | --- |
| Month 1 | Month 2 |  |  |
| *Leucotaraxis* *piniperda* (2022-2025) | | | |
| January | February | -0.5767 | 0.95 |
| January | March | 0.1401 | 1.0 |
| January | April | -0.2928 | 0.995 |
| January | May | 0.1485 | 1.0 |
| February | March | 0.7167 | 0.573 |
| February | April | 0.2838 | 0.976 |
| February | May | 0.7252 | 0.642 |
| March | April | -0.4329 | 0.793 |
| March | May | 0.0.0085 | 1.0 |
| April | May | 0.4414 | 0.852 |

**Table S4:** Post-hoc Tukey’s HSD pairwise comparison of *Leucotaraxis piniperda* collection month, from 2022-2025.

| Pairwise comparison | | Sample size | | Test statistic | *p* value | |
| --- | --- | --- | --- | --- | --- | --- |
| Month 1 | Month 2 | Month 1 | Month 2 |  | Adjusted | Non-adjusted |
| January | February | 9 | 50 | -0.0145 | 1.0 | 0.97 |
| January | March | 9 | 55 | 0.9488 | 1.0 | 0.29 |
| January | April | 9 | 34 | 0.1915 | 1.0 | 0.957 |
| January | May | 9 | 28 | -1.5443 | 0.735 | 0.103 |
| February | March | 50 | 55 | 1.7728 | 0.534 | 0.052* |
| February | April | 50 | 34 | 0.3466 | 1.0 | 0.866 |
| February | May | 50 | 28 | -2.4847 | 0.104 | **0.036** |
| March | April | 55 | 34 | -1.2347 | 1.0 | **0.0**3 |
| March | May | 55 | 28 | -4.0184 | **<0.001** | **<0.001** |
| April | May | 34 | 28 | -2.6001 | 0.084 | **0.005** |

**Table S5:** Results of the post-hoc Dunn test pairwise comparison of *Laricobius nigrinus* prepupae collections by month from 2020-2025. Sample size refers to the number of pupation containers in the comparison. Adjusted (Holm-Bonferroni) and non-adjusted p-values are provided. *Marginal non-adjusted significance.

| Pairwise comparison | | Survival | | Odds ratio | Z ratio | *p* value |
| --- | --- | --- | --- | --- | --- | --- |
| Month 1 | Month 2 | Month 1 | Month 2 |  |  |  |
| February | March | 0.383 ± 0.027 | 0.578 ± 0.014 | 0.453 ± 0.057 | -6.306 | **<0.001** |
| February | April | 0.383 ± 0.027 | 0.546 ± 0.018 | 0.516 ± 0.067 | -5.069 | **<0.001** |
| February | May | 0.383 ± 0.027 | 0.45 ± 0.048 | 0.76 ± 0.17 | -1.228 | 0.609 |
| March | April | 0.578 ± 0.014 | 0.546 ± 0.018 | 1.138 ± 0.103 | 1.422 | 0.486 |
| March | May | 0.578 ± 0.014 | 0.45 ± 0.048 | 1.676 ± 0.34 | 2.548 | 0.053 |
| April | May | 0.546 ± 0.018 | 0.45 ± 0.048 | 1.437 ± 0.387 | 1.847 | 0.251 |

**Table S6:** Pairwise comparisons of *Laricobius nigrinus* survival to adult by collection month from 2020-2025.

| Pairwise comparison | | Survival | | Odds ratio | Z ratio | *p* value |
| --- | --- | --- | --- | --- | --- | --- |
| Year 1 | Year 2 | Year 1 | Year 2 |  |  |  |
| 2020 | 2021 | 0.353 ± 0.029 | 0.0372 ± 0.018 | 0.92 | -0.561 | 0.981 |
| 2020 | 2022 | 0.353 ± 0.029 | 0.454 ± 0.018 | 0.655 | -2.889 | **0.032** |
| 2020 | 2023 | 0.353 ± 0.029 | 0.67 ± 0.019 | 0.269 | -8.536 | **<0.001** |
| 2020 | 2024 | 0.353 ± 0.029 | 0.679 ± 0.02 | 0.258 | -8.604 | **<0.001** |
| 2021 | 2022 | 0.0372 ± 0.018 | 0.454 ± 0.018 | 0.712 | -3.173 | **0.013** |
| 2021 | 2023 | 0.0372 ± 0.018 | 0.67 ± 0.019 | 0.292 | -10.517 | **<0.001** |
| 2021 | 2024 | 0.0372 ± 0.018 | 0.679 ± 0.02 | 0.28 | -10.449 | **<0.001** |
| 2022 | 2023 | 0.454 ± 0.018 | 0.67 ± 0.019 | 0.41 | -7.817 | **<0.001** |
| 2022 | 2024 | 0.454 ± 0.018 | 0.679 ± 0.02 | 0.393 | -7.848 | **<0.001** |
| 2023 | 2024 | 0.67 ± 0.019 | 0.679 ± 0.02 | 0.959 | -0.331 | 0.997 |

**Table S7:** Pairwise comparisons of *Laricobius nigrinus* annual survival to adult.
